# Supplementary material for: Accessing parity-forbidden d-d transitions for photocatalytic CO2 reduction driven by infrared light
Source: Nat Commun. 2023 Jul 7;14:4034. doi: 10.1038/s41467-023-39666-0 (PMC10328996; doi:10.1038/s41467-023-39666-0)
Supplement: Supplementary file 1 — Supplementary Information [file 41467_2023_39666_MOESM1_ESM.pdf]

Supplementary Information

Accessing parity-forbidden *d-d* transitions for photocatalytic CO_2 reduction driven by infrared light

Xiaodong Li[1†], Li Li[2†], Guangbo Chen[3], Xingyuan Chu[3], Xiaohui Liu[3], Chandrasekhar Naisa[3], Darius Pohl[4], Markus Löffler[4] and Xinliang Feng[1,3*]

[1]Max Planck Institute of Microstructure Physics, Weinberg 2, Halle 06120, Germany
[2]Hefei National Laboratory for Physical Sciences at Microscale, University of Science and Technology of China, Hefei, P. R. China
[3]Faculty of Chemistry and Food Chemistry & Center for Advancing Electronics Dresden (cfaed), Dresden University of Technology, Dresden 01062, Germany
[4]Dresden Center for Nanoanalysis (DCN), Dresden University of Technology, Helmholtzstreet, Dresden 01069, Germany
[†]These authors contributed equally: Xiaodong Li, Li Li. *Corresponding author. Email: Xinliang.Feng@tu-dresden.de

Supplementary Figures

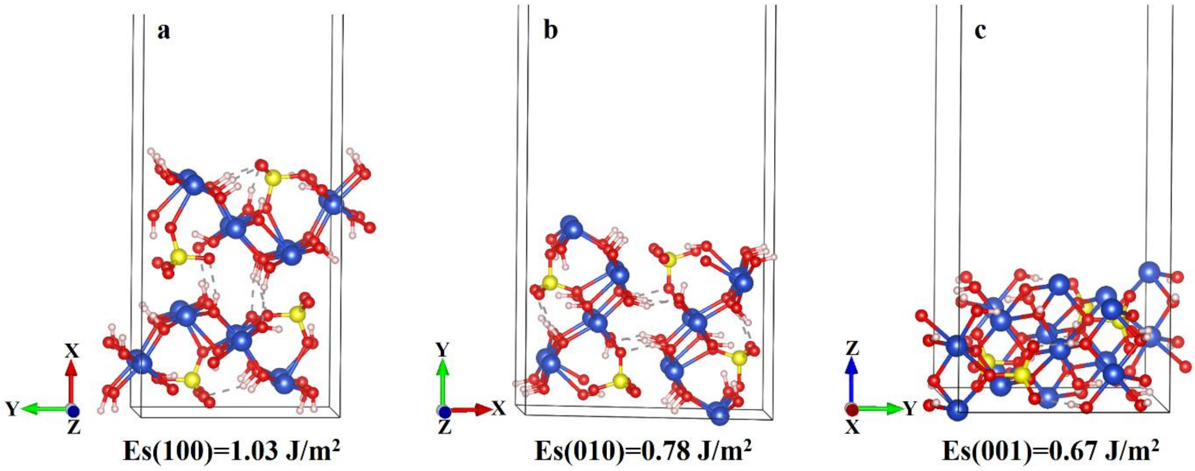

Supplementary Figure 1. The slab models with the thickness of a single unit cell and the corresponding theoretical surface energy for CSON. (a) [100] facet, (b) [010] facet and (c) [001] facet.

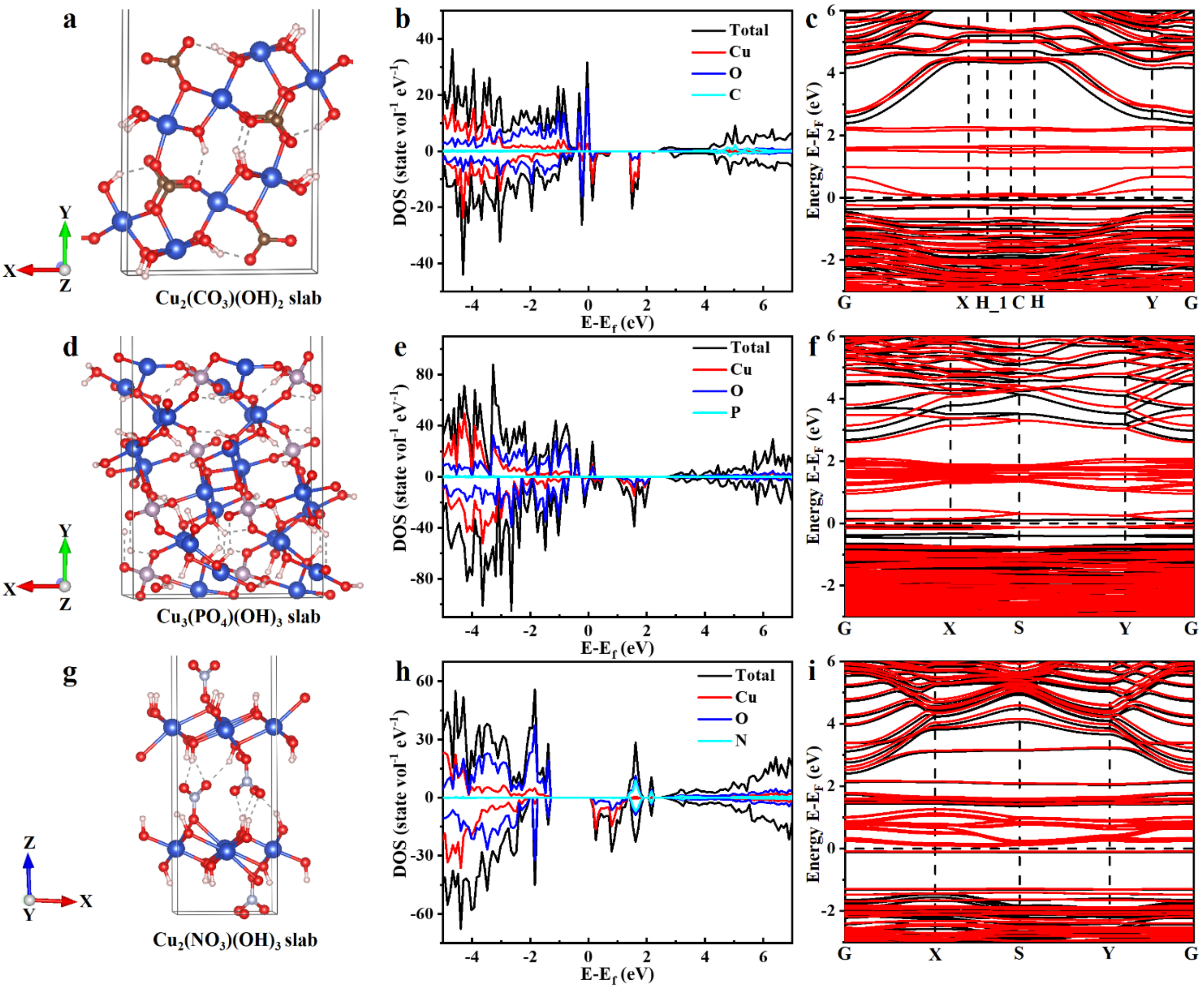

Supplementary Figure 2. Theoretical simulations of 2D CHHS. Theoretical slab models, the density of states and energy band structures for (a)-(c) CCON, (d)-(f) CPON and (g)-(i) CNON. Black and red lines in (c), (f) and (i) represent the spin up and spin down energy band respectively.

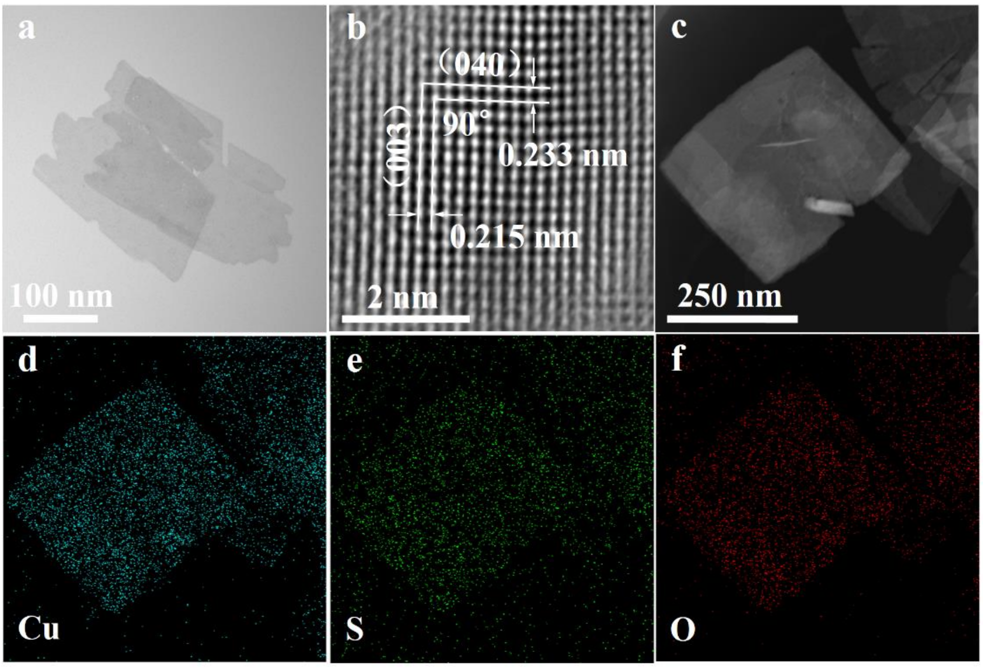

Supplementary Figure 3. Characterizations of p-CSON. (a) TEM image; (b) HRTEM image, in which the exposed facet can be inferred along [100] direction according to the 2D distribution of crystal plane; (c)-(f) annular dark-field TEM images and corresponding elemental mapping images.

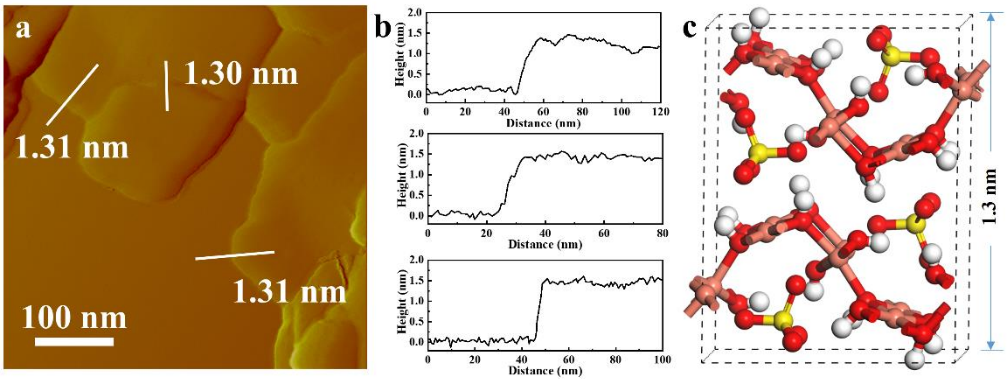

Supplementary Figure 4. AFM Characterizations of p-CSON. (a) AFM image; (b) the corresponding height profiles; (c) theoretical model for p-CSON with the thickness of singe-unit-cell along [100] direction.

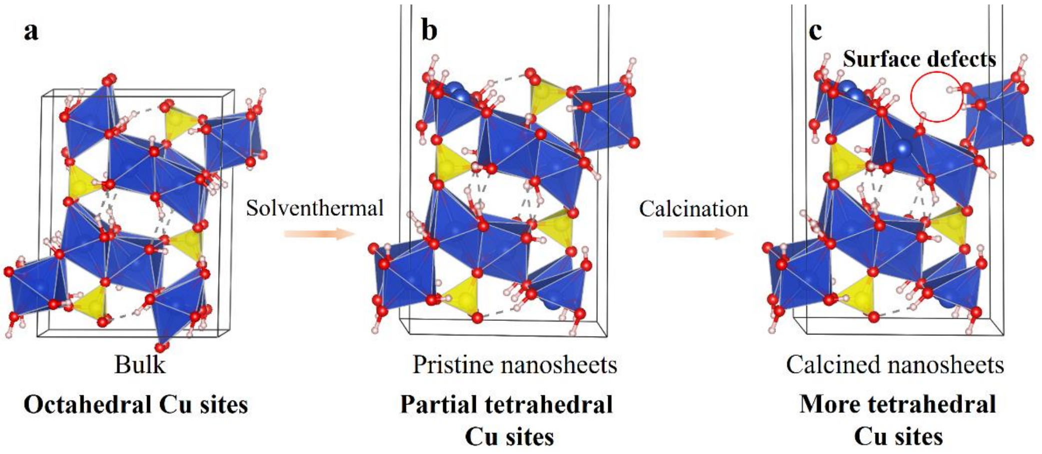

Supplementary Figure 5. Different structure models of $Cu_4(SO_4)(OH)_6$ crystal. (a) $Cu_4(SO_4)(OH)_6$ bulk, (b) p-CSON and (c) c-CSON. In bulk structure, all Cu sites are octahedral, while the Cu sites are partially tetrahedral in pristine nanosheets and much more tetrahedral Cu sites exist in the calcined nanosheets due to the low coordinated environment. For a typical spin-allowed, but Laporte (orbitally) forbidden transition, extinction coefficients for tetrahedral complexes are expected to be around 50-100 times larger than that for the octahedral counterpart. Therefore, more tetrahedral Cu sites can exhibit stronger absorption capability for IR light in hydrotalcite-like hydroxy salts with *d-d* orbital transition.

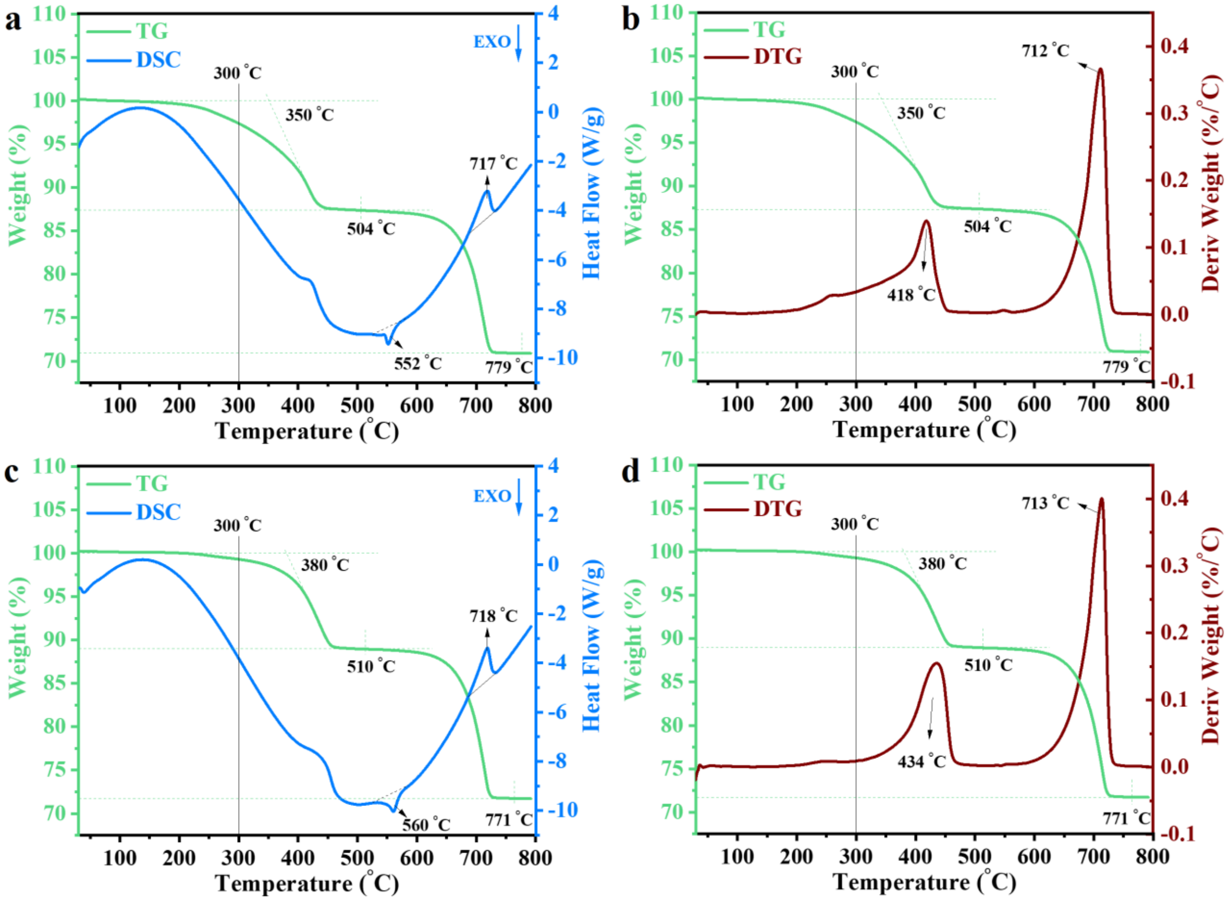

Supplementary Figure 6. (a) TG-DSC and (b) TG-DTG for p-CSON. (c) TG-DSC and (d) TG-DTG for c-CSON.

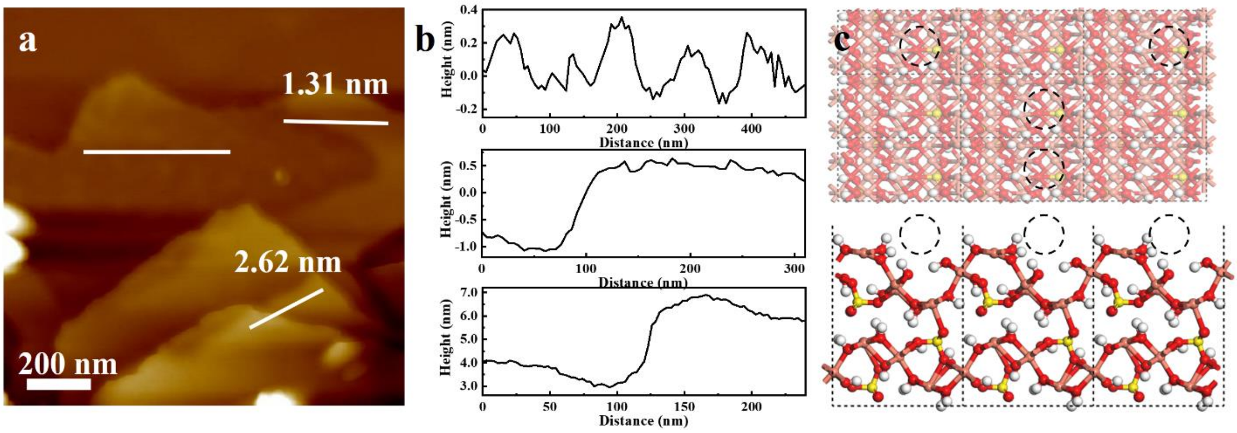

Supplementary Figure 7. AFM characterizations of the c-CSON. (a) AFM image; (b) the corresponding height profiles, in which the first one shows that a defected surface was formed after the calcination; (c) theoretical model for the c-CSON with the thickness of singe-unit-cell along [100] direction, in which the black circle represents the surface defects.

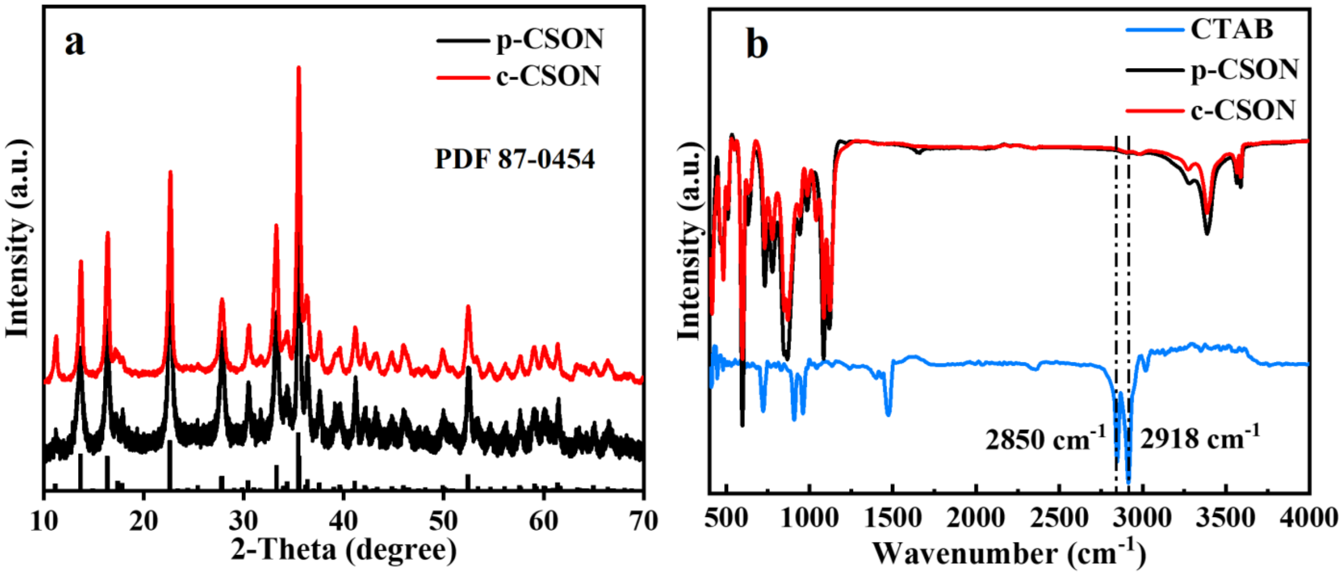

Supplementary Figure 8. Characterizations of p-CSON and c-CSON. (a) XRD patterns and (b) FTIR spectra for p-CSON and c-CSON. One can clearly see that the phase and structures are well retained after the calcination. And both p-CSON and c-CSON possess clean surface without CTAB.

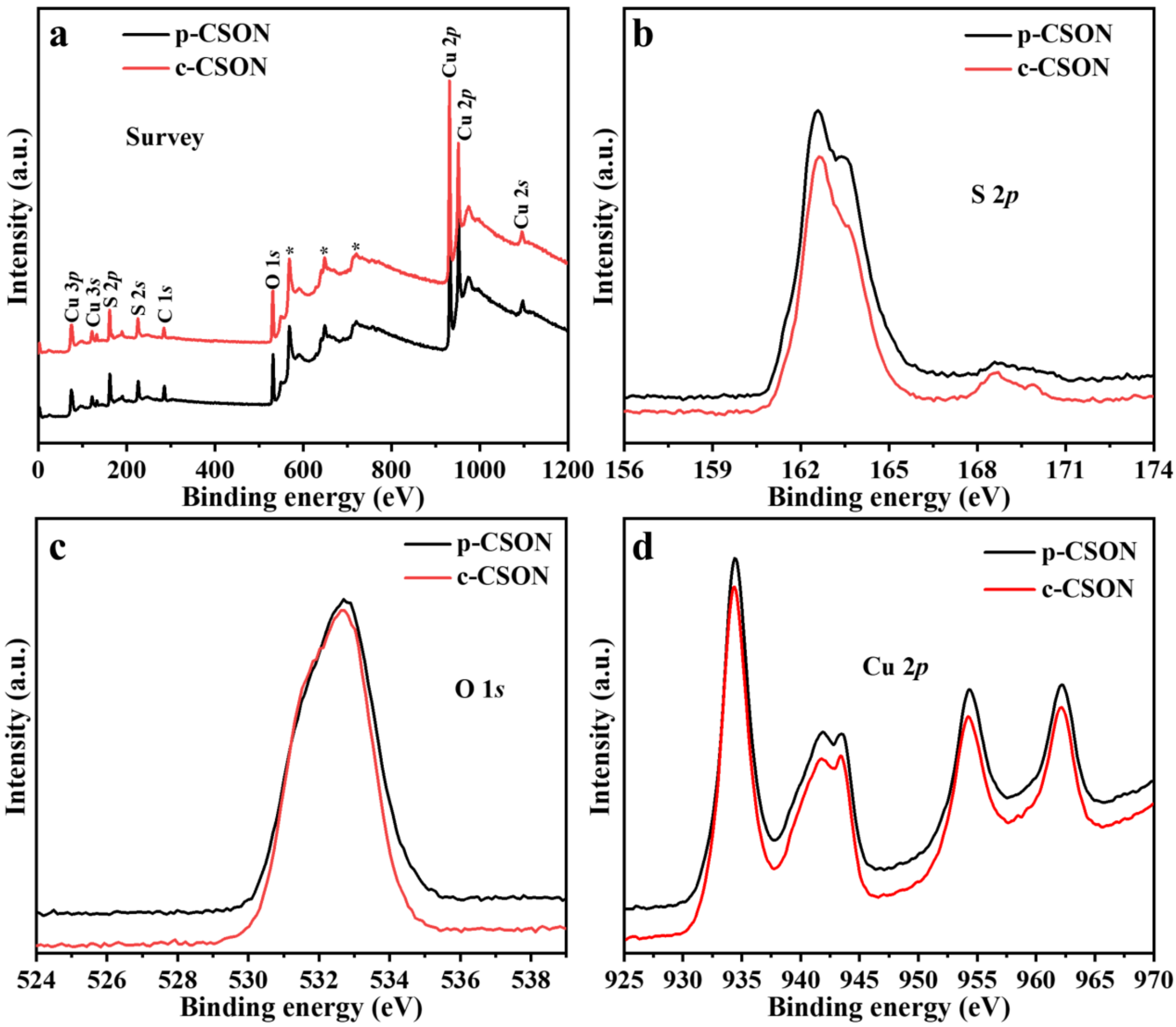

Supplementary Figure 9. The X-ray photoelectron spectra (XPS) for ultrathin 2D CSON. (a) The survey spectra, (b) high-resolution S 2*p* spectra, (c) high-resolution O 1*s* spectra and (d) high-resolution Cu 2*p* spectra for p-CSON (black) and c-CSON (red). The XPS peaks in p-CSON and c-CSON remain almost unchanged.

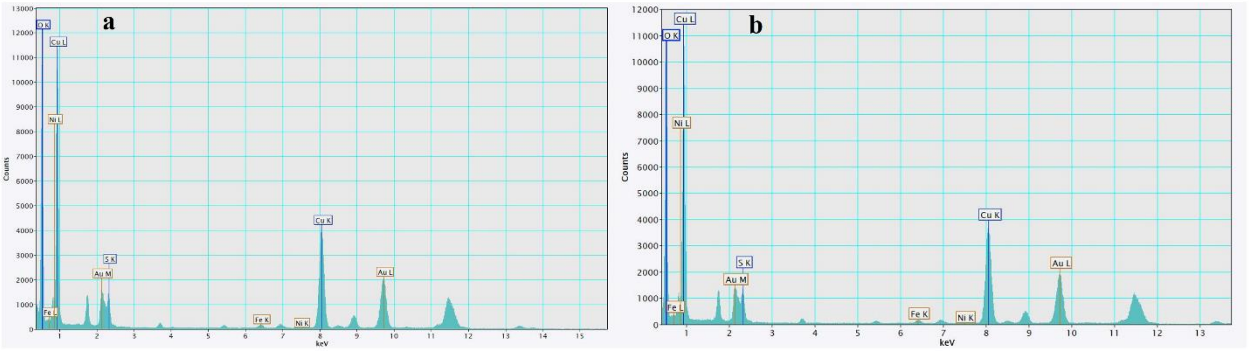

c	EDS element content of TEM								
	**Element**	**Shell**	**Signal (Counts)**	**Fitting Mode**	**Comp. (at.%)**	**Comp. (wt.%)**	**k-Factor (/Si)**	**k-Factor Mode**	**Det. Corr.**
p-CSON	O	K	69.8e+03	Theoretical	63.4	32.67	1.251	Theoretical	0.79
	**S**	**K**	**19.26e+03**	**Theoretical**	**7.42**	**7.66**	**1.063**	**Theoretical**	**0.93**
	Cu	K	88.9e+03	Theoretical	29.16	59.67	1.794	Theoretical	1.0
c-CSON	O	K	53.2e+03	Theoretical	64.7	33.51	1.251	Theoretical	0.79
	**S**	**K**	**11.78e+03**	**Theoretical**	**6.07**	**6.31**	**1.063**	**Theoretical**	**0.93**
	Cu	K	66.6e+03	Theoretical	29.25	60.18	1.794	Theoretical	1.0

Supplementary Figure 10. Energy dispersive spectroscopy (EDS) for ultrathin 2D CSON. (a) p-CSON and (b) c-CSON, in which Au grids were used for these elements mapping and EDS tests; (c) the corresponding atomic percentage in p-CSON and c-CSON by TEM-EDS. Herein, we take the content of S element as a reference for defects because the signal of O could come from the air or the adsorbed O_2, which is generally difficult to preclude from the samples.

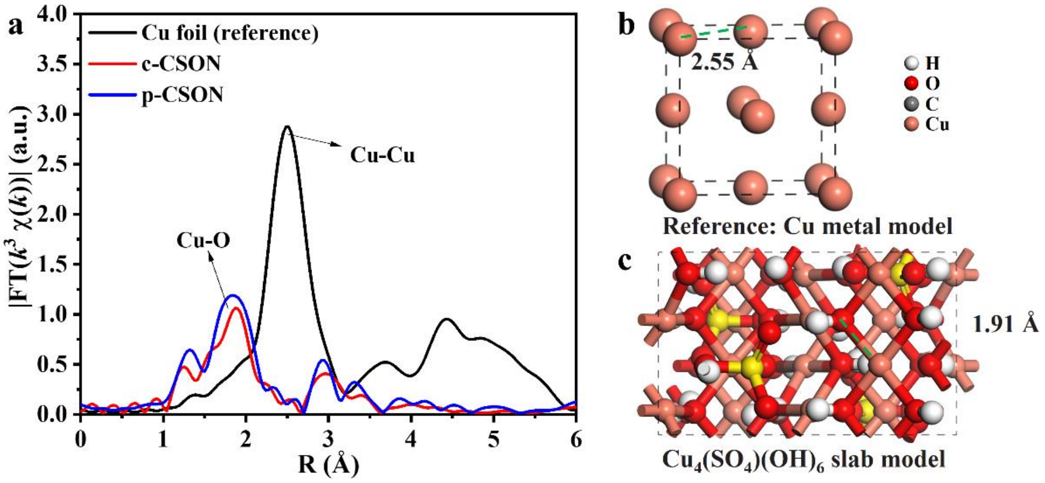

Supplementary Figure 11. (a) Fourier transform (FT) of the Cu K-edge EXAFS spectra. (b) Theoretical Cu metal model. (c) Theoretical $Cu_4(SO_4)(OH)_6$ slab model. The obtained Cu-Cu scattering path of Cu foil from the EXAFS spectra is around 2.51 Å, well-consistent with the theoretical value (2.55 Å) of Cu metal model. And the obtained Cu-O scattering path of both p-CSON and c-CSON from the EXAFS spectra is around 1.89 Å, which is similar with the theoretical value (1.91 Å) of

$Cu_4(SO_4)(OH)_6$ slab model, confirming the reasonability of our theoretical models.

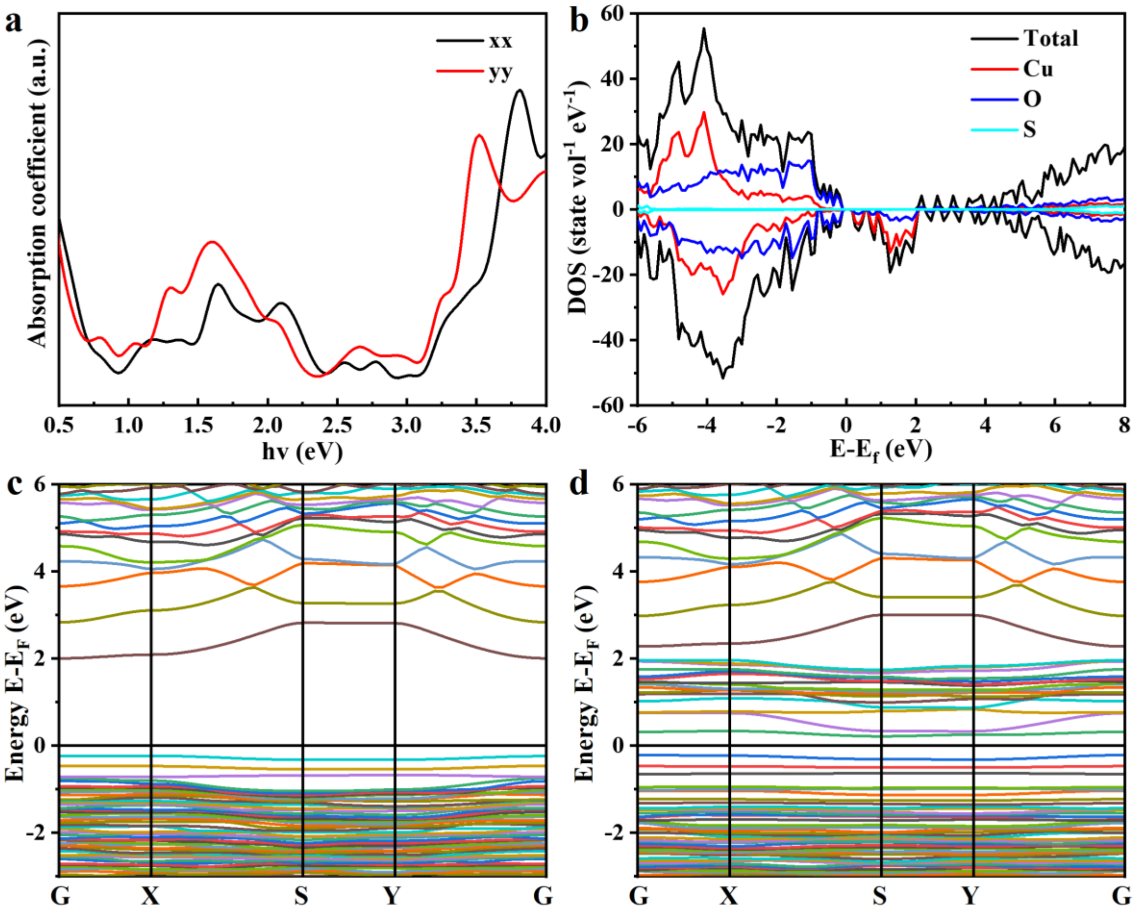

Supplementary Figure 12. (a) The simulated optical absorption spectra, xx and yy represents x and y direction, respectively; (b) density of states; (c) spin-up band structure and (d) spin -down band structure of c-CSON. In (c) and (d), the black lines at y = 0 represent the Fermi level.

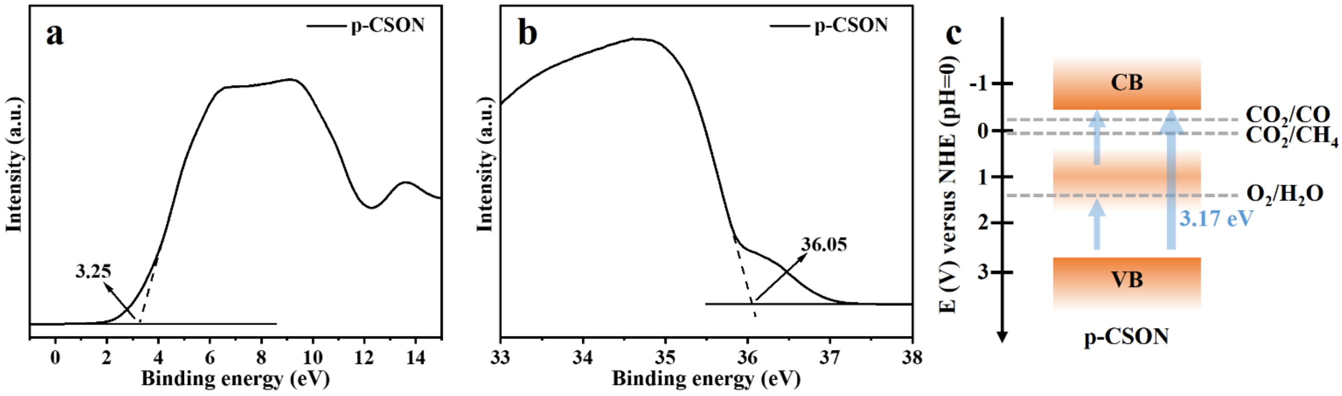

Supplementary Figure 13. (a) Synchrotron-radiation photoemission spectroscopy (SRPES) valence-band, (b) secondary electron cutoff spectra and (c) the corresponding energy band structure with the band edge position for p-CSON.

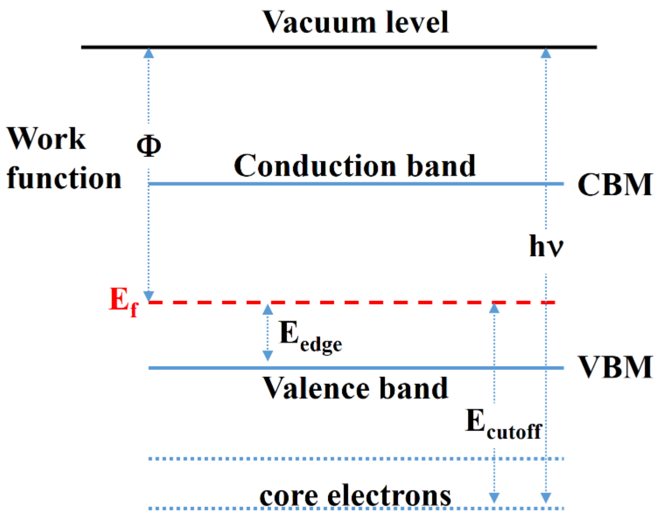

Supplementary Figure 14. Schematic illustration of the energy level information from UPS. The valence band maxima (VBM) of the samples referenced to Normal Hydrogen Electrode (NHE) can be obtained according to the following equations [1-2]:

$$\Phi = h\nu - E_{cutoff}$$

$$E_{VBM} = E_{edge} + \Phi - 4.5 \quad (vs\ \text{NHE, pH} = 0)$$

where Φ is the work function, hν is the photon energy of the excitation source, E_{cutoff} is the energy of secondary electron cutoff, and E_{VBM} is the valence band maxima of samples *vs* NHE at pH = 0.

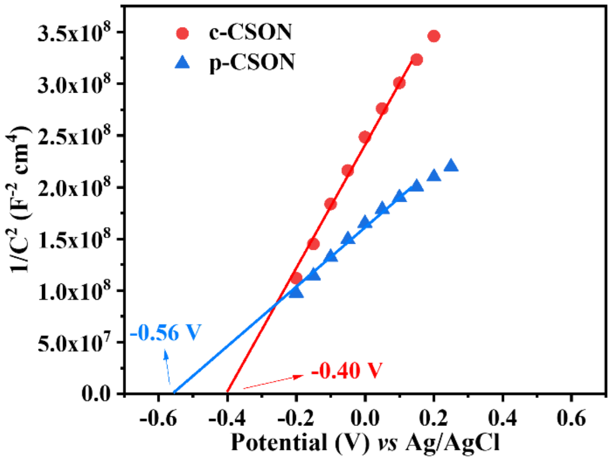

Supplementary Figure 15. Mott-Schottky plots of p-CSON and c-CSON. The Mott-Schottky plots were performed on the CHI 660E electrochemical workstation via a standard three-electrode system in 0.2 M Na_2SO_4 solution which contains a working electrode, a platinum plate as counter electrode. The catalyst (5 mg) was dispersed into a solution of 25 μL 5 wt% Nafion and 0.5 mL isopropanol. Then the resulting mixture (0.2 mL) was deposited onto the surface of FTO and left in the air for drying to prepare the working electrode. The Mott-Schottky plots were recorded at frequency of 1000 Hz. The flat potentials (E_{fb}) of p-CSON and c-CSON are -0.56 V and -0.40 V vs Ag/AgCl (-0.36 V and -0.2 V vs NHE), respectively. Since the conduction band position (E_{CB}) is more negative by ca. 0.1 V than E_{fb} for n-type semiconductor[3-5], the E_{CB} of p-CSON and c-CSON can be calculated to -0.46 V and -0.30 V vs NHE at pH = 0, respectively. According to the intrinsic band gap obtained by the UV-vis-NIR diffuse reflectance

spectra, we can also get the valence band position (E_{VB}) of p-CSON and c-CSON to 2.71 V and 2.43 V, respectively. The obtained band structures by the Mott-Schottky plots are quite similar to that of UPS.

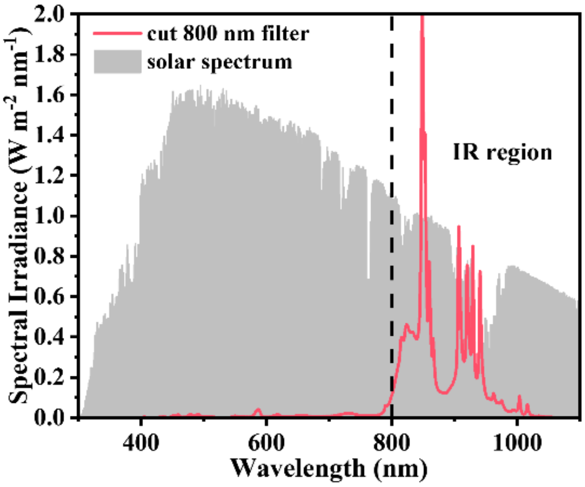

Supplementary Figure 16. The illumination spectrum of our light simulator comparing with sunlight.

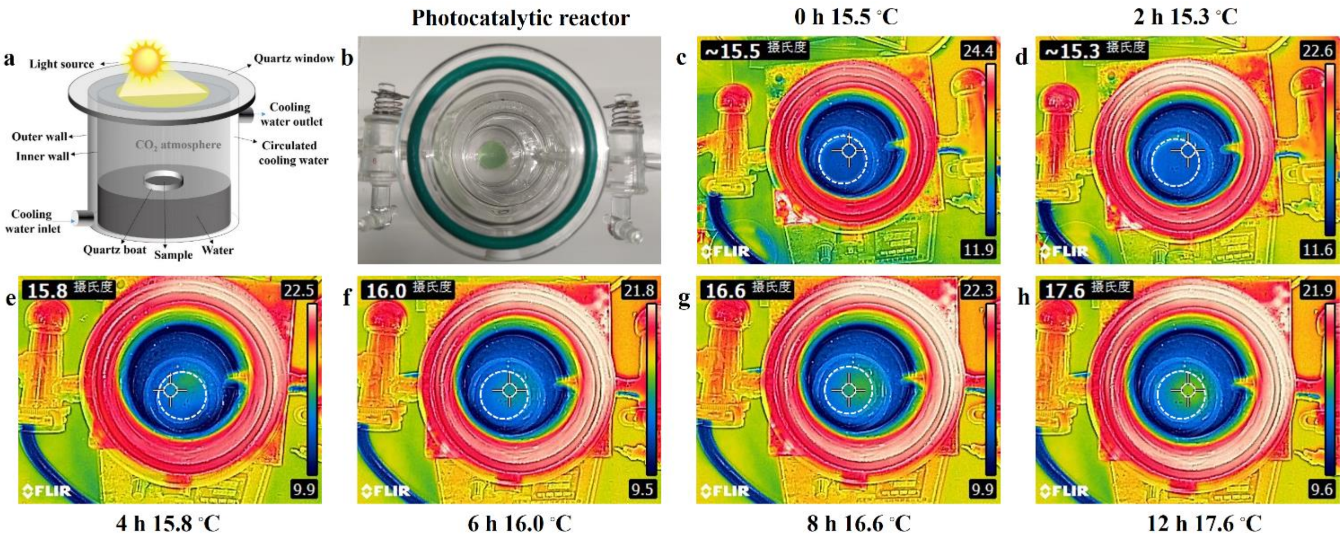

Supplementary Figure 17. (a) Schematic diagram and (b) photograph of the photocatalytic reactor. (c)-(h) *In situ* thermographic photographs during photocatalysis. Thermographic photographs measured by FLIR E8. The dotted white circles indicate the location of the c-CSON-based thin film, and the provided temperatures correspond to the average temperatures of the c-CSON-based thin film during IR light irradiation.

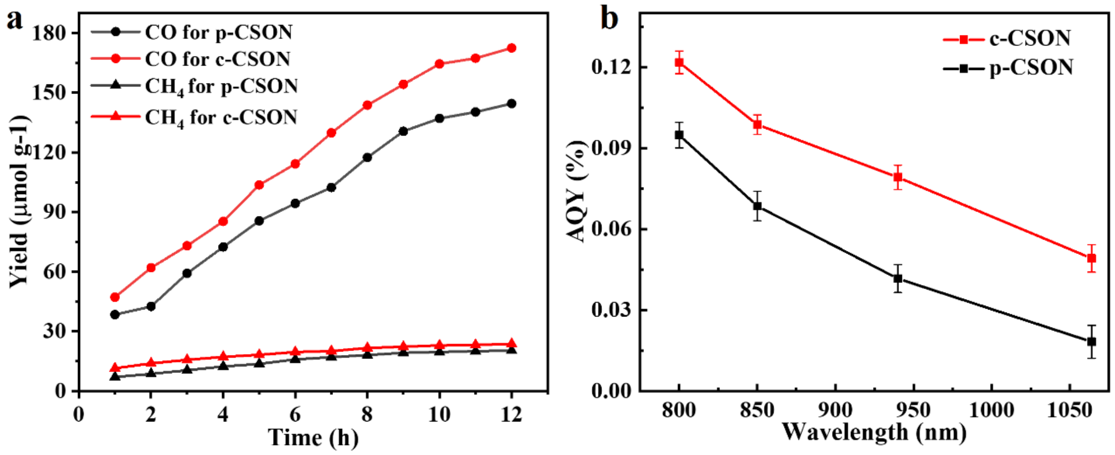

Supplementary Figure 18. Performance of ultrathin 2D CSON for IR light-driven CO_2 reduction. (a) Products of photocatalytic CO_2 reduction for p-CSON (black) and c-CSON (red) under IR light irradiation within 12 hours; (b) Apparent quantum yield (%) for p-CSON (black) and c-CSON (red). The number of effective electrons was determined by CO yields of 12 h photoreaction under a monochromatic light wavelength at 800, 850, 940 and 1064 nm.

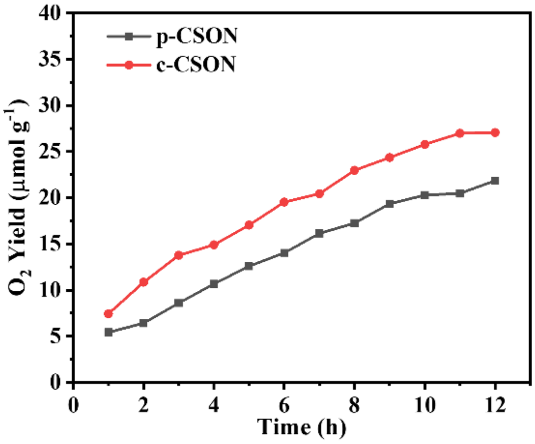

Supplementary Figure 19. Performance evaluation for oxidation half reaction. O_2 yield for p-CSON (black) and c-CSON (red) under IR light-driven CO_2 reduction within 12 hours.

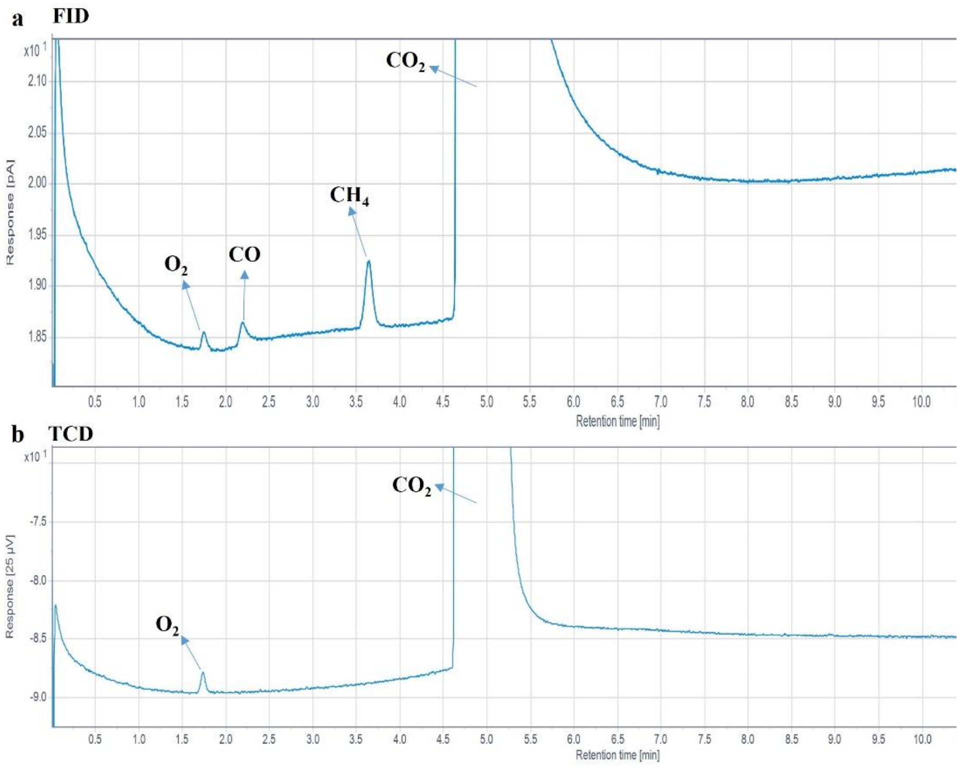

Supplementary Figure 20. The detected gas products by GC using c-CSON as the catalyst after 2 h IR-light irradiation. The gas products were quantified by the Agilent GC-8860 gas chromatograph equipped with a TDX-01 column. (a) A flame ionization detector (FID) and (b) a thermal conductivity detector (TCD).

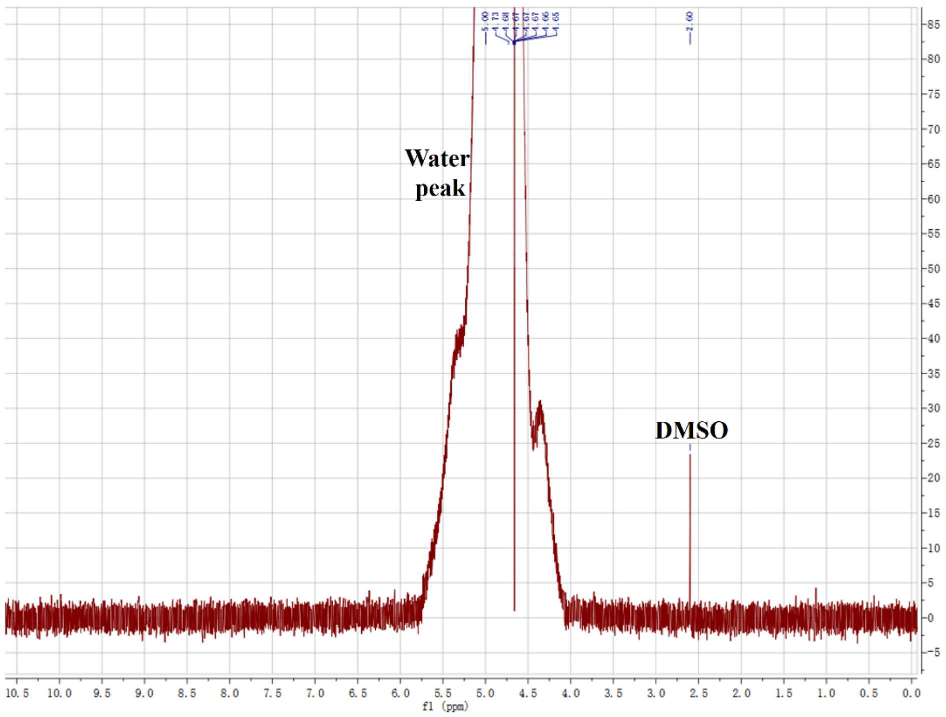

Supplementary Figure 21. Representative ^{1}H NMR spectrum of the liquids after 12 h IR light-driven

CO_2 photocatalysis over c-CSON. The liquid products were quantified by nuclear magnetic resonance (NMR) (Bruker AVANCE AV III 400) spectroscopy, in which dimethyl sulfoxide (DMSO, Sigma, 99.99%) was used as the internal standard.

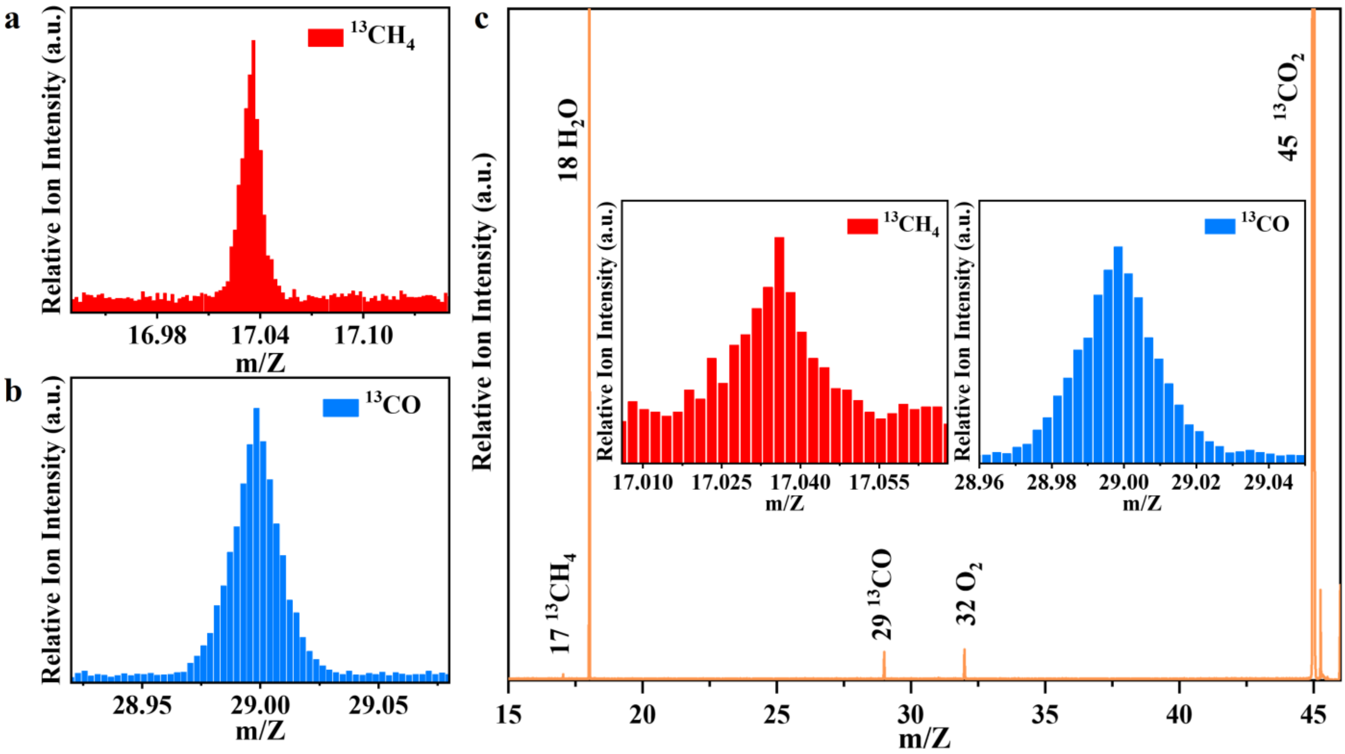

Supplementary Figure 22. Synchrotron-based vacuum ultraviolet photoionization mass spectrometry (SVUV-PIMS) spectrum of the products after $^{13}CO_2$ photoreduction for (a)-(b) c-CSON and (c) p-CSON at *hv* = 14.5 eV. Insets of (c): signals of m/z = 17 ($^{13}CH_4$) and m/z = 29 (^{13}CO), detected at photon energies, *hv* = 14.5 eV.

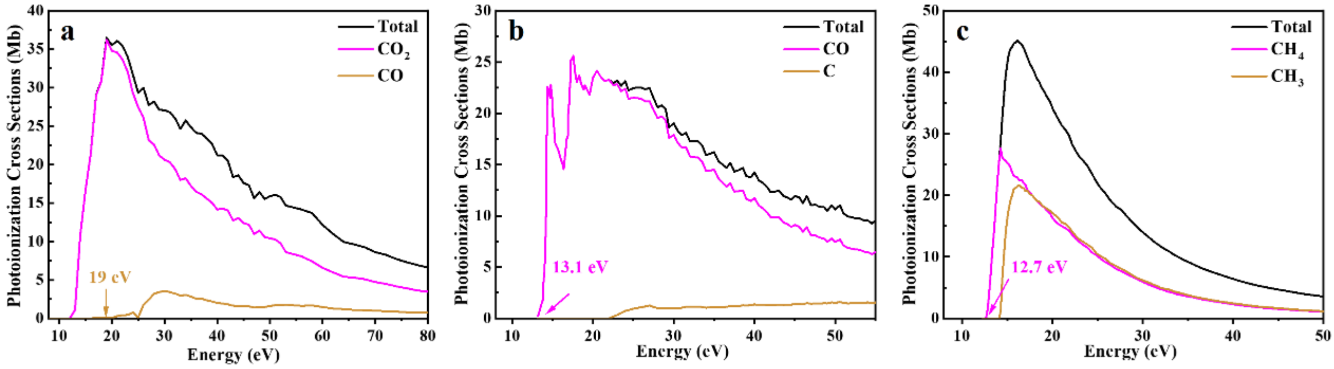

Supplementary Figure 23. Synchrotron-based vacuum ultraviolet photoionization mass spectrometry (SVUV-PIMS). Absolute photoionization cross sections for (a) CO_2, (b) CO and (c) CH_4. *http://flame.nsrl.ustc.edu.cn/database/data.php* **Supplementary Figure** 23a reveals that CO_2 would dissociate into CO when the photon energy approaches about 19 eV; meanwhile, the pure CO and CH_4 can be detected when the photon energy is up to 13.1 eV and 12.7 eV, respectively (**Supplementary**

Figure 23b-c). As such, it is feasible to utilize SVUV-PIMS spectra at the photon energy of 14.5 eV for distinguishing whether CO and CH_4 is obtained from CO_2 reduction or dissociation.

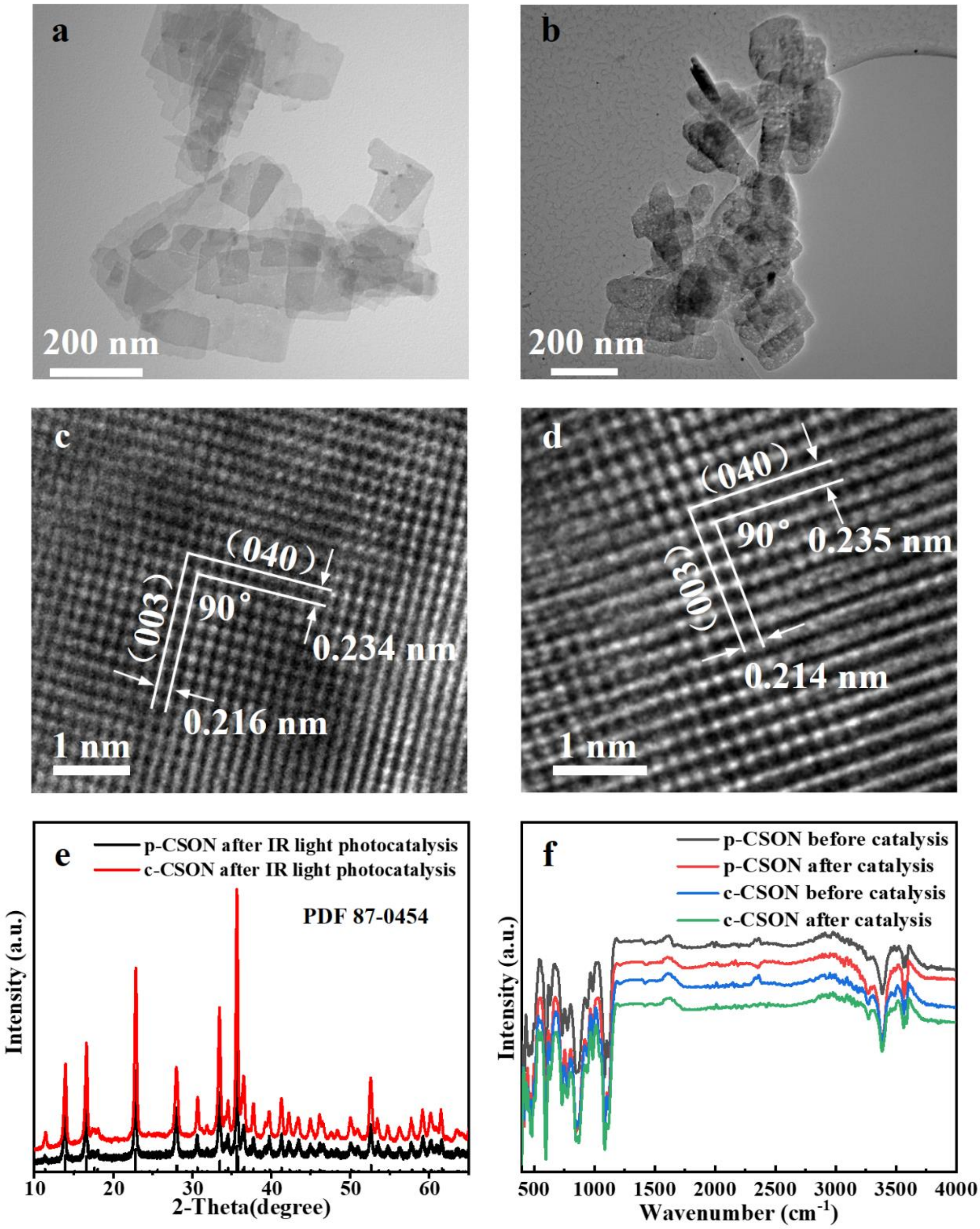

Supplementary Figure 24. Characterizations for p-CSON and c-CSON after the continuous 96 h test for photocatalytic CO_2 under IR light irradiation. TEM images for (a) p-CSON and (b) c-CSON. HRTEM images for (c) p-CSON and (d) c-CSON. XRD patterns (e) and FTIR spectra (f) for p-CSON and c-CSON.

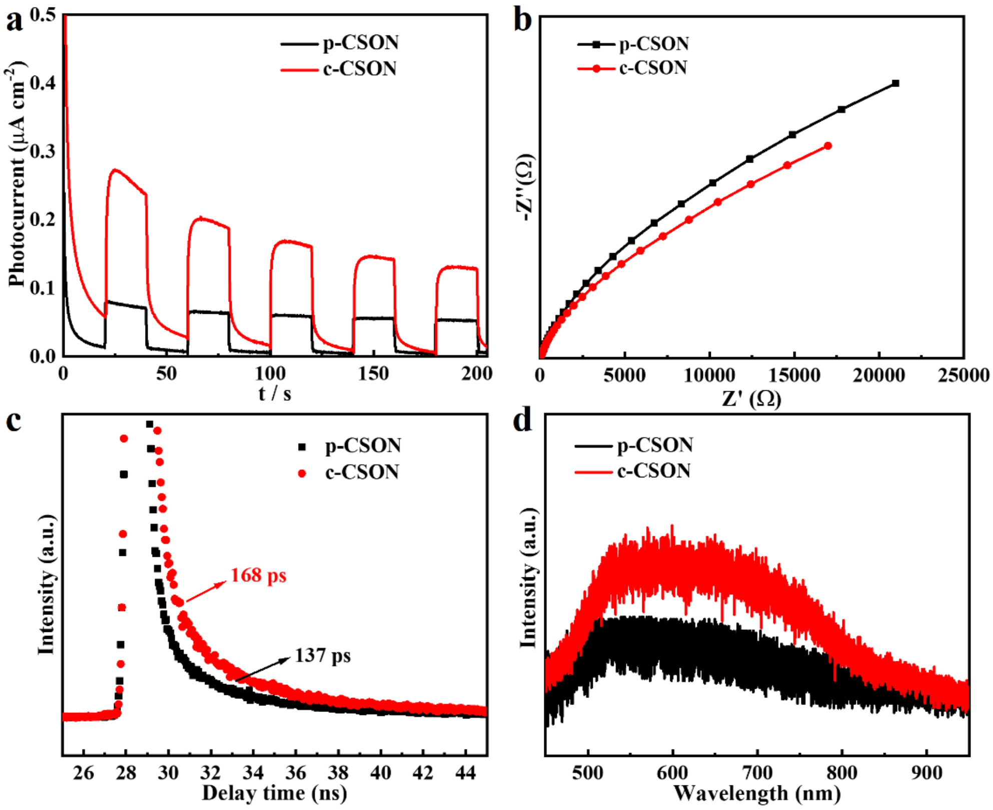

Supplementary Figure 25. (a) Transient photocurrent response spectra, (b) Nyquist plots of electrochemical impedance spectroscopy, (c) fluorescence emission decay spectra and (d) room-temperature photoluminescence (PL) spectra for p-CSON and c-CSON.

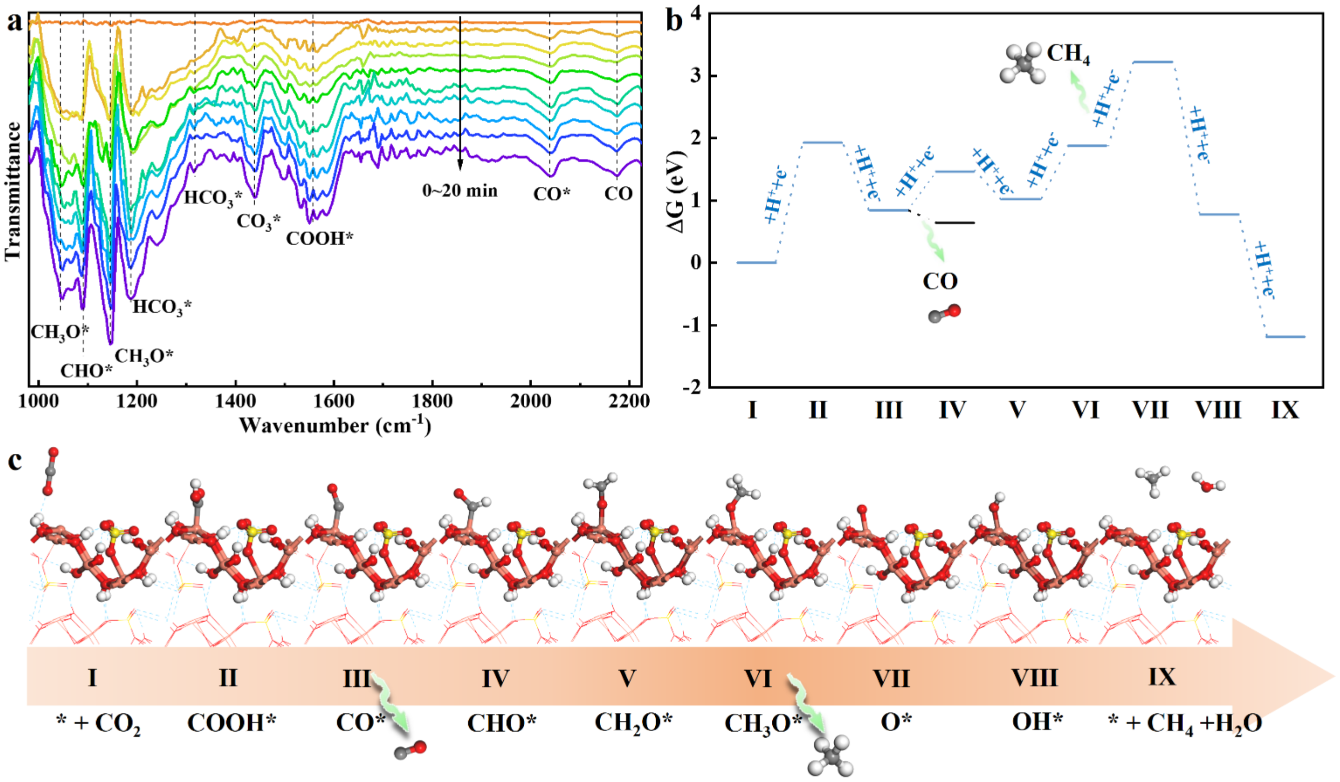

Supplementary Figure 26. Mechanistic studies of ultrathin 2D p-CSON for IR light-driven CO_2 reduction. (a) *In situ* FTIR spectroscopy characterization, (b) Gibbs free energy diagrams and (c) intermediate structure of p-CSON during CO_2 reduction under IR light irradiation.

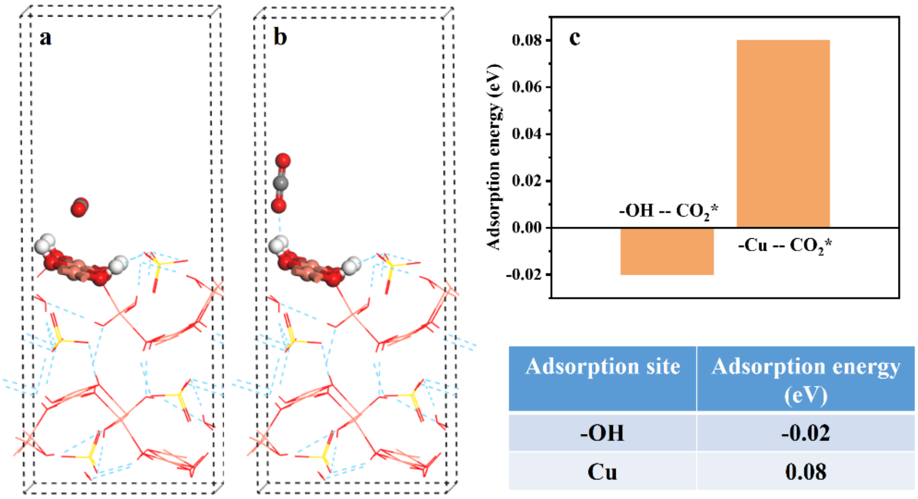

Adsorption site	Adsorption energy (eV)
-OH	-0.02
Cu	0.08

Supplementary Figure 27. CO_2 adsorption on the surface of ultrathin 2D CSON. CO_2 adsorption models on the (a) Cu sites and (b) OH sites for p-CSON. (c) The table show the CO_2 adsorption energy for Cu and OH sites.

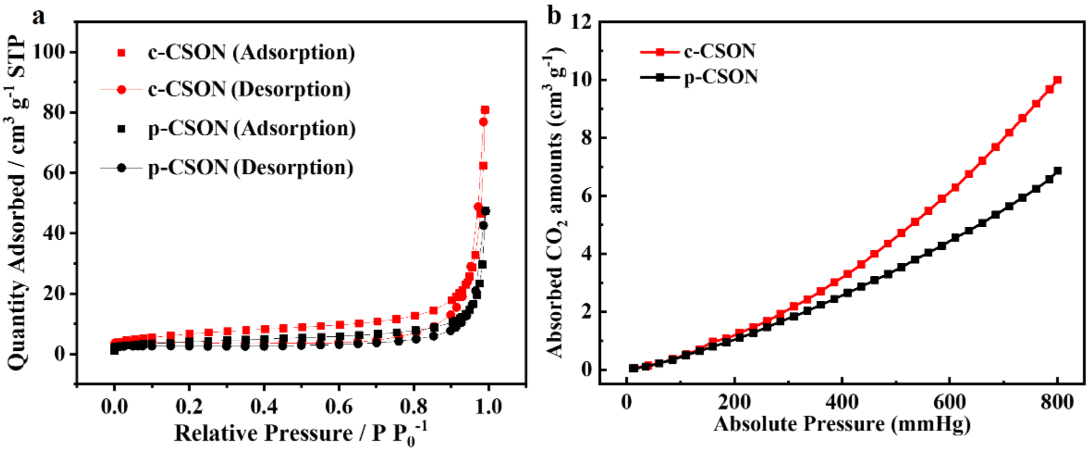

Supplementary Figure 28. (a) BET isotherms and (b) CO_2 adsorption isotherms for p-CSON and c-CSON. The BET surface area for c-CSON is 24. 2 m^2 g^{-1}, which is around 2 times than that of p-CSON (14.4 m^2 g^{-1}), fairly agreeing with the corresponding CO_2 adsorption isotherms.

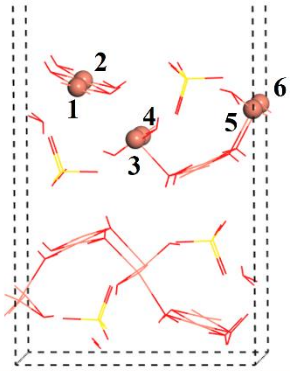

Cu	1	2	3	4	5	6
Pristine model	-1.048874	-1.049672	-1.14083	-1.118972	-1.133581	-1.121178
Calcined model	-0.962954	-0.94928	-1.021477	-0.882621	-1.018029	-1.041739

Supplementary Figure 29. The 2D slab model of p-CSON and corresponding Bader charges in surface Cu atoms. The numbers (1-6) insert mean the different Cu sites. The table below shows the calculated Bader charge in different Cu sites of p-CSON and c-CSON. As we can see, the surface Cu atoms in c-CSON transfer fewer electrons away, suggesting their higher charge density than that in the pristine one.

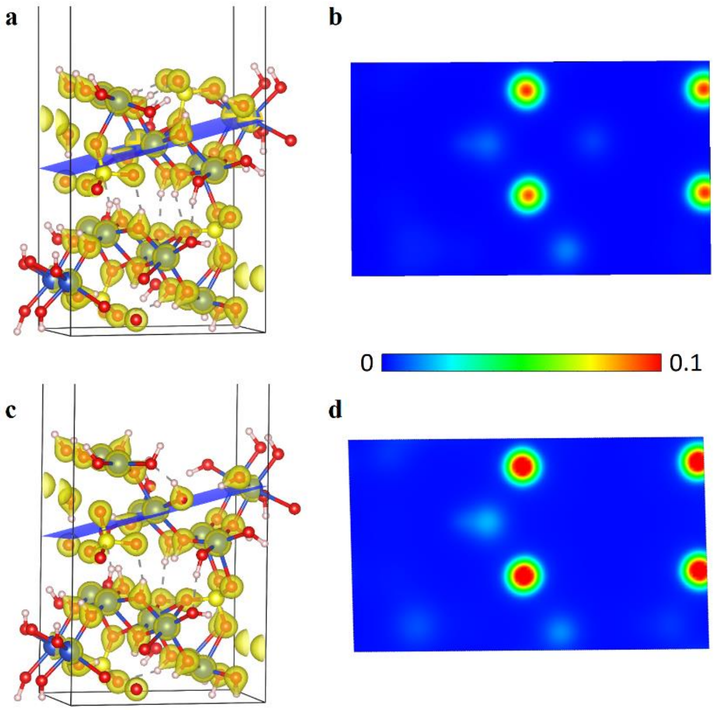

Supplementary Figure 30. 3D and 2D distribution of charge density. (a)-(b) p-CSON and (c)-(d) c-CSON in Cu atoms near the -SO_4 vacancies, in which the state density of c-CSON is much larger than that of the pristine one, well consistent with the result of Bader charge.

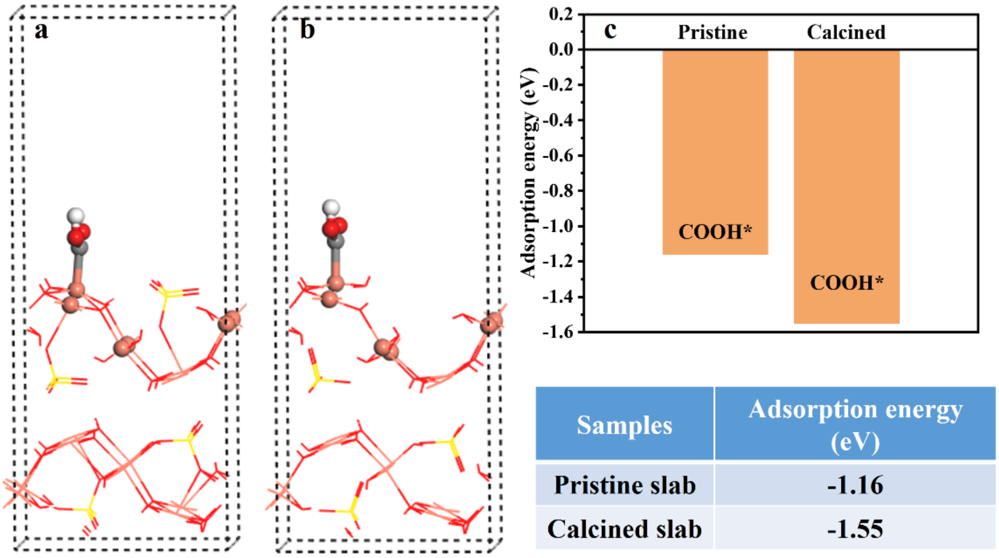

Samples	Adsorption energy (eV)
Pristine slab	-1.16
Calcined slab	-1.55

Supplementary Figure 31. The adsorption models of the key intermediate (COOH*) in the rate-determining step during the CO_2 photoreduction for (a) p-CSON and (b) c-CSON. The corresponding adsorption energy is shown in figure (c) and the below table.

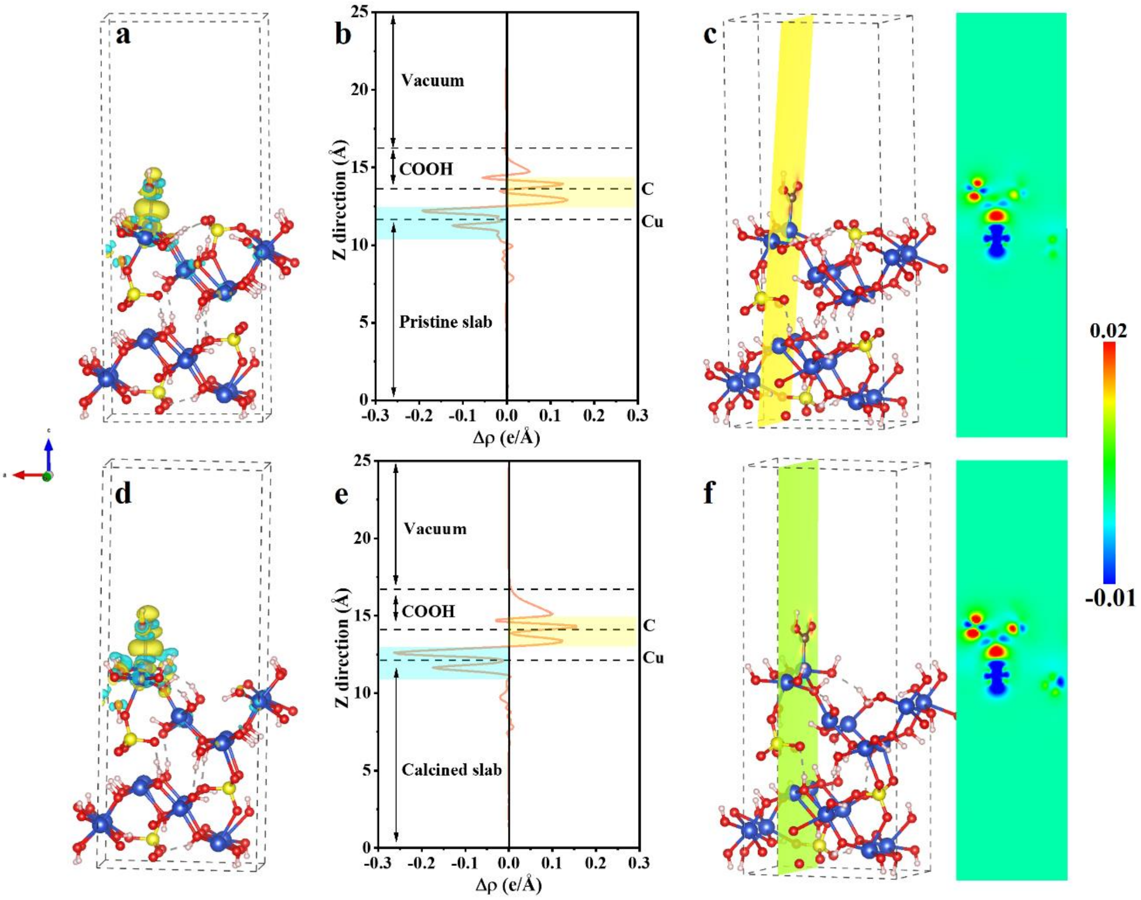

Supplementary Figure 32. The charge density difference for COOH* intermediate in (a)-(c) p-CSON and (d)-(f) c-CSON. The yellow and green isosurfaces correspond to the increase in the number of electrons and the depletion zone, respectively. The isosurfaces are 0.002 e $Bohr^{-3}$.

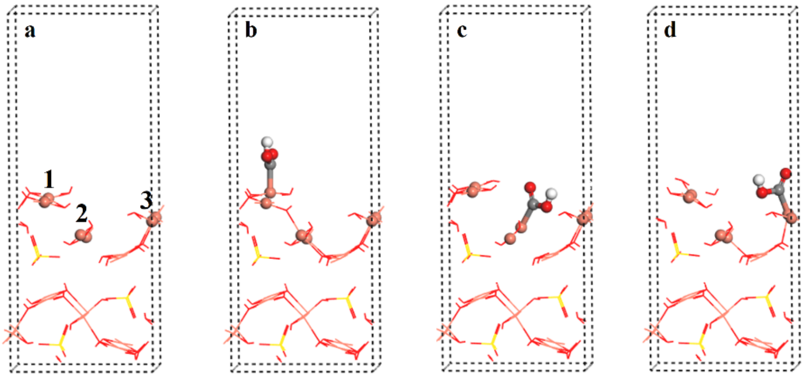

Active site	Calculated energy (eV)	Adsorption energy (eV)
-COOH	-24.04	/
1	-422.22	-1.55
2	-422.17	-1.50
3	-422.05	-1.38

Supplementary Figure 33. COOH* adsorption models on different Cu sites in c-CSON. (a) The slab model of c-CSON with different Cu sites (1-3) on the surface. (b)-(d) The COOH* adsorption models on the 1, 2, 3 Cu sties respectively. The below table shows the specific energy of the models and corresponding adsorption energy by DFT calculations.

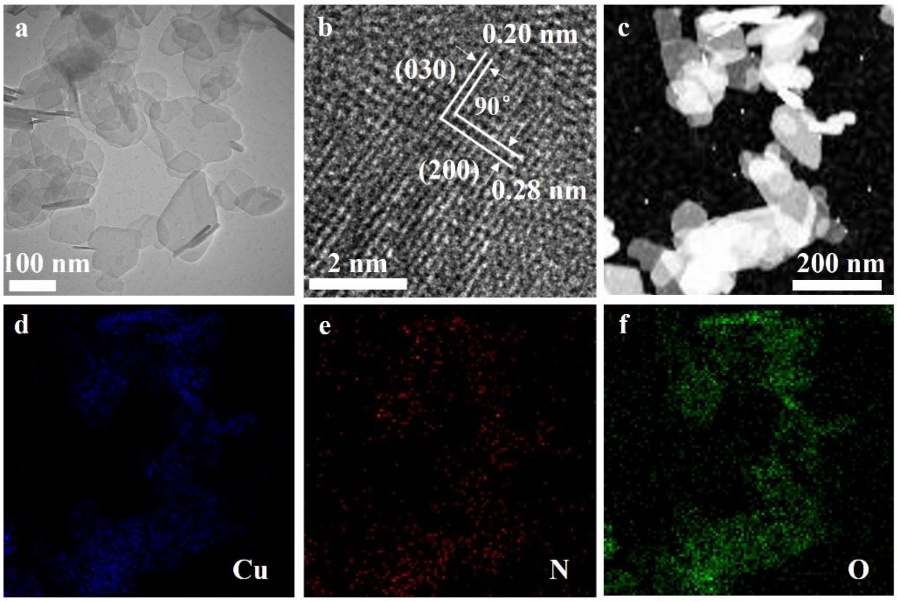

Supplementary Figure 34. Characterizations of CNON. (a) TEM image; (b) HRTEM image, in which the exposed facet can be inferred along [001] direction according to the 2D distribution of crystal plane; (c)-(f) annular dark-field TEM images and corresponding elemental mapping images.

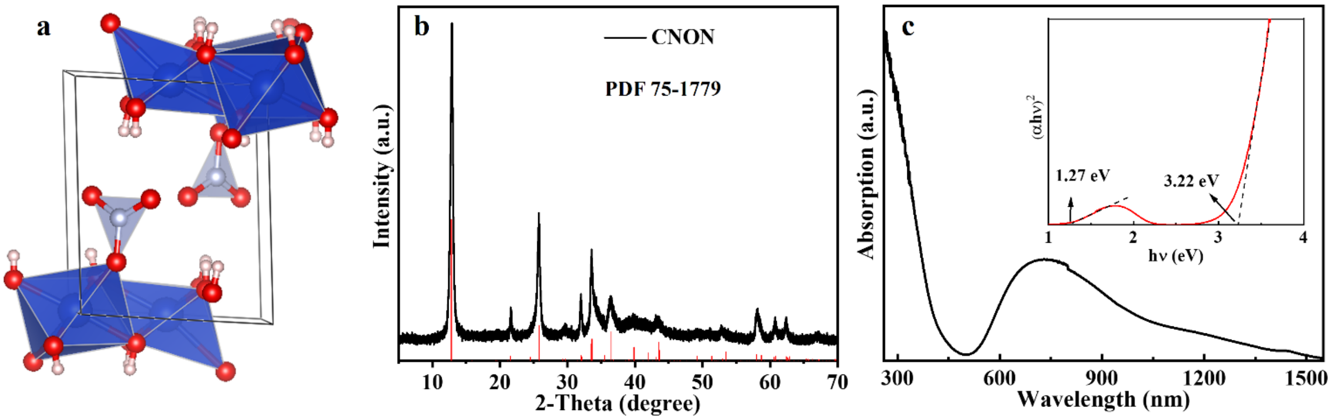

Supplementary Figure 35. (a) Theoretical model, (b) XRD pattern and (c) UV-vis-NIR diffuse reflectance spectra (insert is the corresponding Tauc plot) for CNON.

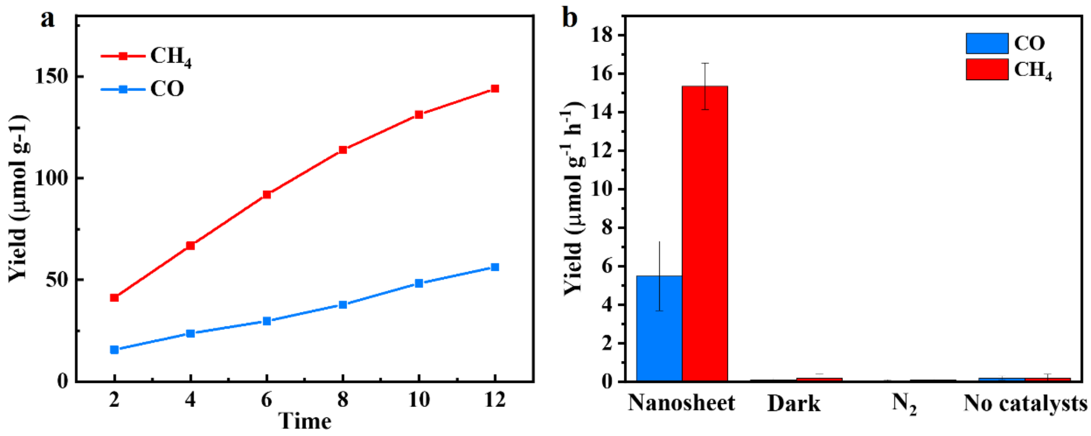

Supplementary Figure 36. Yields of photocatalytic CO_2 reduction to CO and CH_4 (a) with reaction time and (b) over different catalytic conditions using CNON, error bars represent the s. d. of three independent measurements.

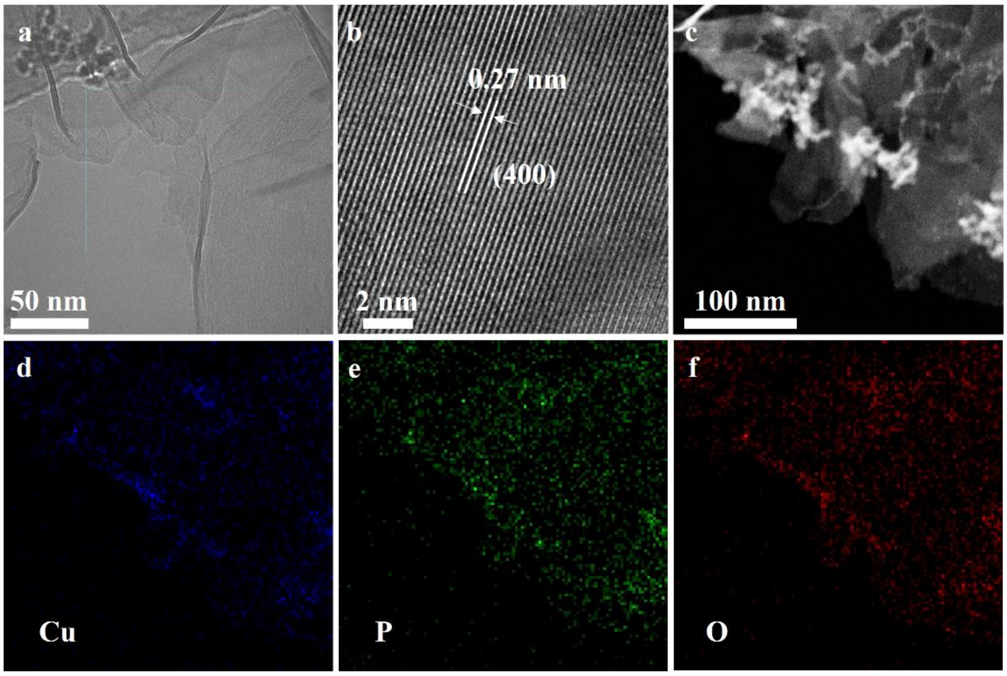

Supplementary Figure 37. Characterizations of CPON. (a) TEM image; (b) HRTEM image; (c)-(f) annular dark-field TEM images and corresponding elemental mapping images.

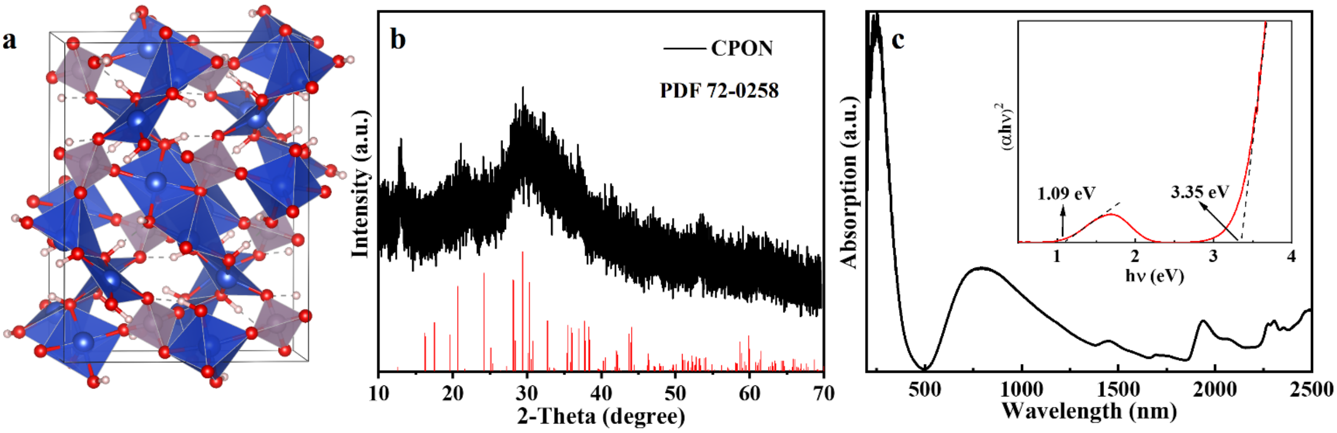

Supplementary Figure 38. (a) Theoretical model, (b) XRD pattern and (c) UV-vis-NIR diffuse reflectance spectra (insert is the corresponding Tauc plot) for CPON.

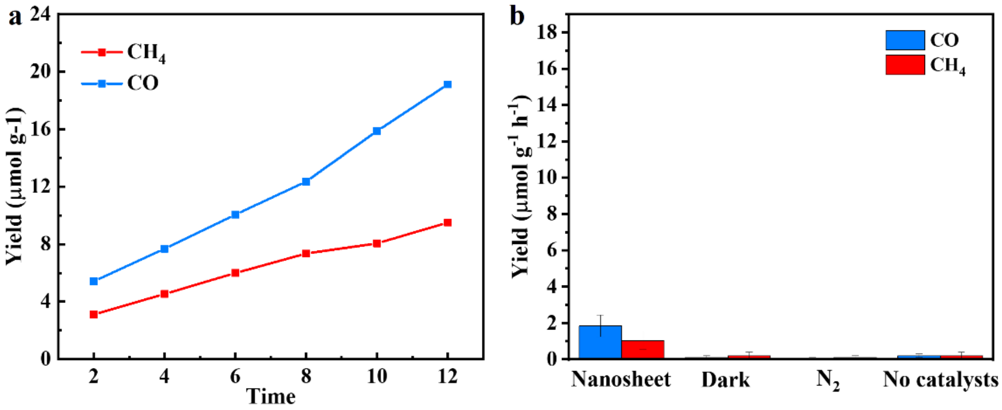

Supplementary Figure 39. Yields of photocatalytic CO_2 reduction to CO and CH_4 (a) with reaction time and (b) over different catalytic conditions using CPON, error bars represent the s. d. of three independent measurements.

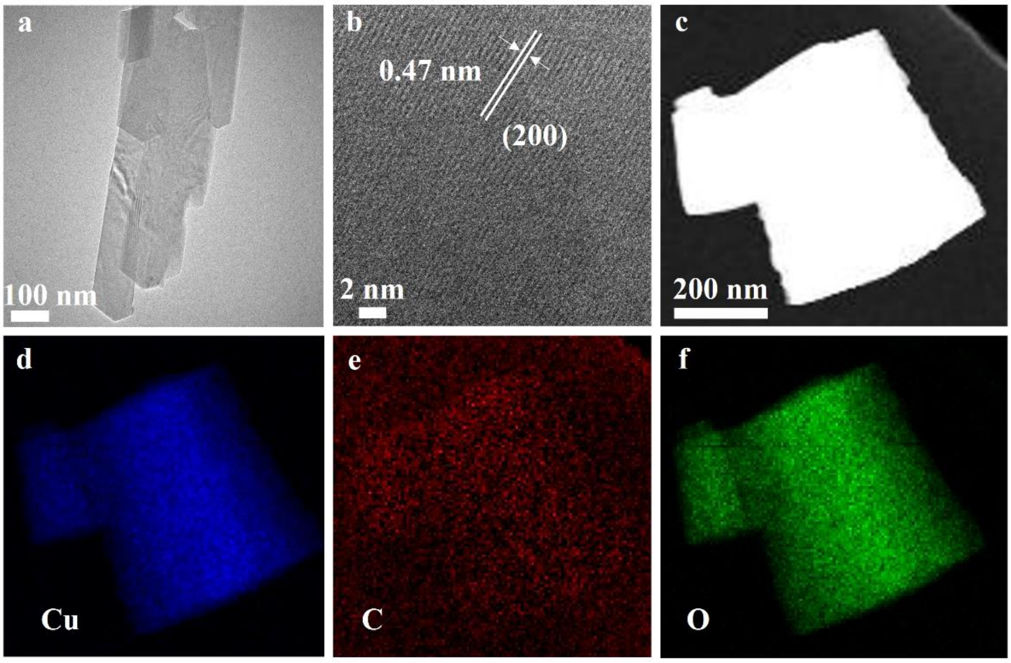

Supplementary Figure 40. Characterizations of CCON. (a) TEM image; (b) HRTEM image; (c)-(f) annular dark-field TEM images and corresponding elemental mapping images.

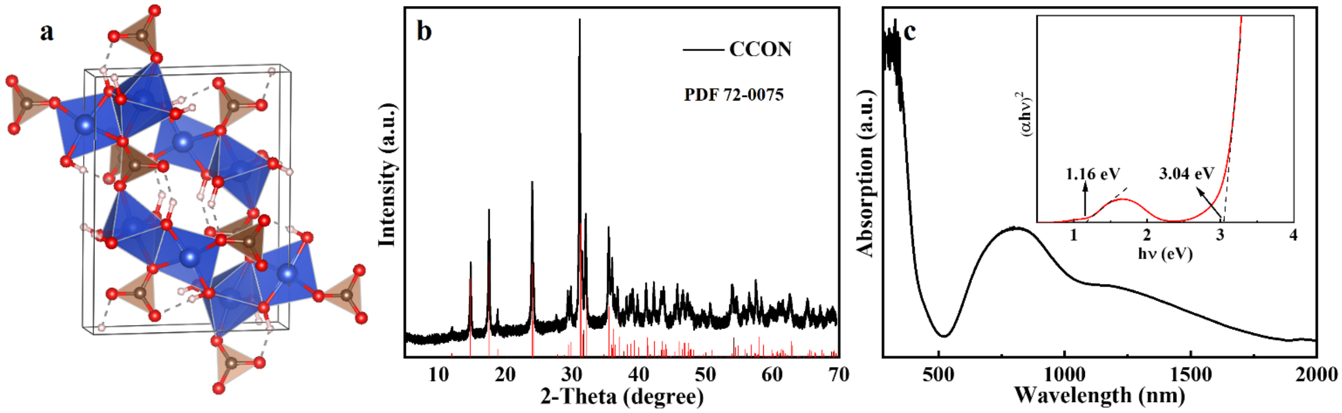

Supplementary Figure 41. (a) Theoretical model, (b) XRD pattern and (c) UV-vis-NIR diffuse reflectance spectra (insert is the corresponding Tauc plot) for CCON.

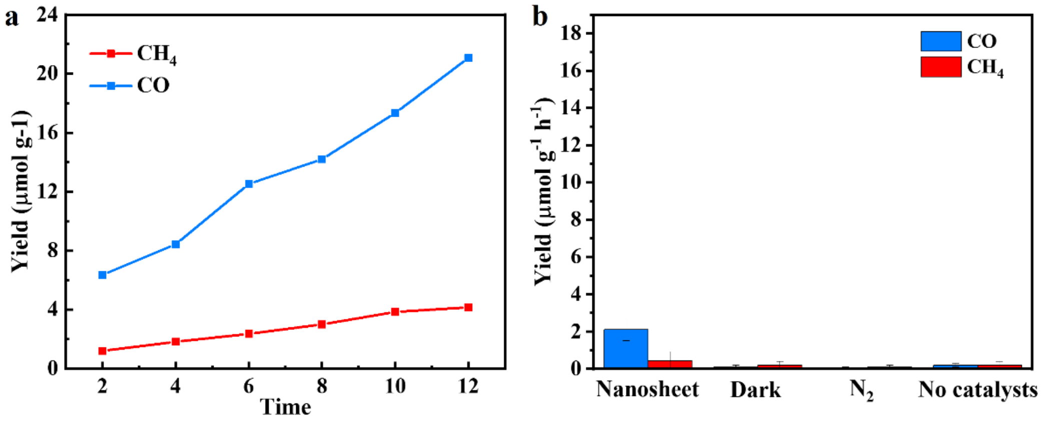

Supplementary Figure 42. Yields of photocatalytic CO_2 reduction to CO and CH_4 (a) with reaction time and (b) over different catalytic conditions using CCON, error bars represent the s. d. of three independent measurements.

Supplementary Tables

Supplementary Table 1. The content of S element in p-CSON and c-CSON determining by the XPS analysis. Herein, we take the content of S element as a reference for defects because the signal of O could come from the air or the adsorbed O_2, which is generally difficult to preclude from the samples.

Species	XPS component of S element
p-CSON	26.7 %
c-CSON	20.6 %

Supplementary Table 2. Comparison of the reaction performances with other single-component catalysts for IR light-driven CO_2 reduction without sacrificial agents. (RT: Room Temperature).

Catalysts	IR Light source	Reaction medium	Products and Activity	Apparent quantum yield (AQY)	Reference
**c-CSON**	**300 W Xe lamp (AM1.5 and Cut800 filter)**	**Gas-solid, water, RT**	**CO (21.95 μmol g^{-1} h^{-1}) and CH_4 (4.11 μmol g^{-1} h^{-1})**	**0.122 % (at 800 nm)**	**This work**
**p-CSON**	**300 W Xe lamp (AM1.5 and Cut800 filter)**	**Gas-solid, water, RT**	**CO (17.73 μmol g^{-1} h^{-1}) and CH_4 (2.97 μmol g^{-1} h^{-1})**	**0.095 % (at 800 nm)**	**This work**
**$Cu_2(NO_3)(OH)_3$ nanosheets**	**300 W Xe lamp (AM1.5 and Cut800 filter)**	**Gas-solid, water, RT**	**CO (5.49 μmol g^{-1} h^{-1}) and CH_4 (15.34 μmol g^{-1} h^{-1})**	**/**	**This work**
**$Cu_3(PO_4)(OH)_3$ nanosheets**	**300 W Xe lamp (AM1.5 and Cut800 filter)**	**Gas-solid, water, RT**	**CO (1.84 μmol g^{-1} h^{-1}) and CH_4 (1.03 μmol g^{-1} h^{-1})**	**/**	**This work**
**$Cu_2(CO_3)(OH)_2$ nanosheets**	**300 W Xe lamp (AM1.5 and Cut800 filter)**	**Gas-solid, water, RT**	**CO (2.11 μmol g^{-1} h^{-1}) and CH_4 (0.43 μmol g^{-1} h^{-1})**	**/**	**This work**
WO_3 nanosheet with oxygen vacancies	40 W silicon nitride lamp	Gas-solid, water, RT	CO (2.70 μmol g^{-1} h^{-1})	0.0274 % (at 800 nm)	[6]
Metallic CuS atomic layers	300 W Xe lamp (AM1.5 and Cut800 filter)	Gas-solid, water, RT	CO (14.5 μmol g^{-1} h^{-1})	0.05 % (at 800 nm)	[7]
CoN porous atomic layers	300 W Xe lamp (AM1.5 and Cut800 filter)	Gas-solid, water, RT	CO (0.29 μmol g^{-1} h^{-1})	/	[8]
Ni-CoS_2 nanosheets	300 W Xe lamp (AM1.5 and Cut800 filter)	Gas-solid, water, RT	CH_4 (101.8 μmol g^{-1} h^{-1})	/	[9]
1 wt.% carbon quantum dots loading Bi_2WO_6 nanosheets	A 500-W xenon arc lamp (Cut700 filter)	Gas-solid, water, RT	CH_4 (0.051 μmol g^{-1} h^{-1})	/	[10]
$Rb_{0.33}WO_3$	300 W Xe lamp (Cut800 filter)	Gas-solid, water, RT	CH_3OH (15.48 μmol g^{-1} h^{-1})	/	[11]
Few-layered BiOI	300 W Xe lamp (Cut700 filter)	Gas-solid, water, RT	CO (0.79 μmol g^{-1} h^{-1})	0.02 % (at 700 nm)	[12]
Defective $Bi_{19}Br_3S_{27}$ nanowires	300 W Xe lamp (λ > 700 nm)	Gas-solid, water, RT	CH_3OH (0.4 μmol g^{-1} h^{-1})	/	[13]

Supplementary Table 3. Total energy (eV) of p-CSON and c-CSON and corresponding intermediates.

Unit (eV)	Slab	COOH*	CO*	CHO*	CH_2O*	CH_3O*	O*	OH*
Pristine model	-427.66	-452.86	-442.71	-445.54	-449.90	-452.85	-430.60	-436.78
Calcined model	-396.63	-422.22	-411.89	-414.74	-418.73	-422.30	-401.63	-406.32

Supplementary Table 4. Free energy (eV) of each step during CO_2 photoreduction for p-CSON and c-CSON.

Unit (eV)	ΔG (*)	ΔG (COOH*)	ΔG (CO*)	ΔG (CO)	ΔG (CHO*)	ΔG (CH_2O*)	ΔG (CH_3O*)	ΔG (O*)	ΔG (OH*)	ΔG (CH_4)
Pristine model	0	1.925	0.84	0.64	1.465	1.02	1.875	3.22	0.775	-1.19
Calcined model	0	1.535	0.63	0.64	1.495	1.16	1.395	1.16	0.205	-1.19

Supplementary Table 5. Free energy (eV) correction for species of p-CSON and c-CSON.

Species	E (eV)	ZPE (eV)	TS (eV)
H_2	-6.76	0.27	0.40
CO_2	-22.98	0.31	0.66
H_2O	-14.21	0.56	0.67
CO	-14.79	0.13	0.6
CH_4	-24.04	1.2	0.6
COOH*	/	0.60	0.25
CO*	/	0.18	0.19
CHO*	/	0.45	0.19
CH_2O*	/	0.72	0.25
CH_3O*	/	1.04	0.21
O*	/	0.04	0.12
OH*	/	0.31	0.10

Supplementary References

1. Liu, F. et al. Direct Z-scheme hetero-phase junction of black/red phosphorus for photocatalytic water splitting. *Angew. Chem. Int. Ed.* **58**, 11791–11795 (2019)
2. Zhao, D. et al. Boron-doped nitrogen-deficient carbon nitride-based Z-scheme heterostructures for photocatalytic overall water splitting. *Nat. Energy* **6**, 388–397 (2021).
3. Xia, YS. et al. Tandem utilization of CO_2 photoreduction products for the carbonylation of aryl iodides. *Nat. Commun.* **13**, 2964 (2022).
4. Yang, M. et al. Photocatalytic cyclization of nitrogen-centered radicals with carbon nitride through promoting substrate/catalyst interaction. *Nat. Commun.* **13**, 4900 (2022).
5. Chakraborty, S. et al. Wurtzite $CuGaS_2$ with an in-situ-formed CuO layer photocatalyzes CO_2 conversion to ethylene with high selectivity. *Angew. Chem.Int. Ed.* **62**, e202216613 (2023).
6. Liang, L. et al. Infrared light-driven CO_2 overall splitting at room temperature. *Joule* **2**, 1004–1016 (2018).
7. Li, X. et al. Ultrathin conductor enabling efficient IR light CO_2 reduction. *J. Am. Chem. Soc.* **141**,

423−430 (2019).
8. Liang, L. et al. Efficient infrared light induced CO_2 reduction with nearly 100% CO selectivity enabled by metallic CoN porous atomic layers. *Nano Energy* **69**, 104421 (2020).
9. Xu, J. et al. Efficient infrared-light-driven CO_2 reduction over ultrathin metallic Ni-doped CoS_2 nanosheets. *Angew. Chem. Int. Ed.* **60**, 8705–8709 (2021).
10. Kong, X. et al. Harnessing vis-NIR broad spectrum for photocatalytic CO_2 reduction over carbon quantum dots-decorated ultrathin Bi_2WO_6 nanosheets. *Nano Res.* **10**, 1720−1731 (2017).
11. Wu, X. et al. Photocatalytic CO_2 conversion of $M_{0.33}WO_3$ directly from the air with high selectivity: insight into full spectrum induced reaction mechanism. *J. Am. Chem. Soc.* **141**, 5267–5274 (2019).
12. Ye, L. et al. Synthesis of olive-green few-layered BiOI for efficient photoreduction of CO_2 into solar fuels under visible/near-infrared light. *Sol. Energy. Mat. Sol. Cells* **144**, 732−739 (2016).
13. Li, J. et al. Interfacial engineering of $Bi_{19}Br_3S_{27}$ nanowires promotes metallic photocatalytic CO_2 reduction activity under near-infrared light irradiation. *J. Am. Chem. Soc.* **143**, 6551−6559 (2021).
